# Ten years of change in clinical disease status and treatment in rheumatoid arthritis: results based on standardized monitoring of patients in an ordinary outpatient clinic in southern Norway

**DOI:** 10.1186/s13075-015-0716-0

**Published:** 2015-08-20

**Authors:** Glenn Haugeberg, Inger Johanne Widding Hansen, Dag Magnar Soldal, Tuulikki Sokka

**Affiliations:** Department of Rheumatology, Hospital of Southern Norway Trust, Servicebox 416, 4604 Kristiansand S, Norway; Division of Rheumatology, Department of Neuroscience, Norwegian University of Science and Technology, Trondheim, Norway; Department of Rheumatology, Jyväskylä Central Hospital, Jyväskylä, Finland

## Abstract

**Introduction:**

In the new millennium, clinical outcomes in patients with rheumatoid arthritis (RA) have improved. Despite a large number of register data, there is a lack of data reflecting the entire outpatient RA population, and in particular long-term data. The main aim of this study was to explore changes in clinical disease status and treatment in an RA outpatient clinic population monitored with recommended outcome measures over a 10-year period.

**Methods:**

Standard data collected included demographic data, erythrocyte sedimentation rate, C-reactive protein, clinical measures of disease activity (Disease Activity Score in 28 joint counts [DAS28], Clinical Disease Activity Index [CDAI], Simplified Disease Activity Index [SDAI] and global assessments) and patient-reported outcomes (measures of physical function, joint pain, fatigue, patient global assessment and morning stiffness). Treatment with disease-modifying antirheumatic drugs (DMARDs) was also recorded, as well as rheumatoid factor (RF) and anti-citrullinated protein antibody (ACPA) status.

**Results:**

In the RA population, the mean age was approximately 64 years and disease duration was 10–12 years. About 70 % were females; approximately 20 % were current smokers; and 65–70 % were positive for RF and ACPA. During follow-up, disease activity improved significantly. When we applied the DAS28, CDAI, SDAI and Boolean criteria for remission, the proportions of patients in remission increased from 21.3 %, 8.1 %, 5.8 % and 3.8 %, respectively, in 2004 to 55.5 %, 31.7 %, 31.8 % and 17.7 %, respectively, in 2013. The proportions of patients with DAS28, CDAI and SDAI low disease activity status were 16.0 %, 34.0 %, and 34.9 %, respectively, in 2004 and 17.8 %, 50.4 % and 50.8 %, respectively, in 2013. A significant improvement in patient-reported outcome was seen only for the full 10-years, but not for the last 4 years, of the study period. The proportion of patients taking synthetic (about 60 %) and biologic (approximately 30 %) DMARDs was stable over the last 4 years of the study period, with no significant change observed, whereas the proportion of patients being treated with prednisolone was reduced significantly from 61 % in 2010 to 54 % in 2013.

**Conclusions:**

The encouraging data we present suggest that the vast majority of patients with RA monitored in outpatient clinics in the new millennium can expect to achieve a status of clinical remission or low disease activity.

## Introduction

The prognosis and clinical outcome in rheumatoid arthritis (RA) has improved significantly over the last 10–20 years. Rheumatology has changed its focus from “care” to “cure,” and “bed” departments have been changed into outpatient clinics. In this period, remission has become a realistic treatment goal [1, 2]. This has been attributed to new treatment options [3] and new treatment strategies [4]. The new treatment options include biologic disease-modifying antirheumatic drugs (DMARDs) [3] and the use of a triple combination of synthetic DMARDs [5]. The new treatment recommendations in RA include early aggressive treatment and treating patients toward remission or to achieve low disease activity if remission is not obtainable [6]. To reach this goal, the use of clinical outcome measures to monitor patients with RA has been advocated by leading rheumatology experts as standard practice in ordinary clinical care [6–8].

With the introduction of biologic DMARDs, leading experts proposed creating patient registers for long-term follow-up to obtain real-life data [9]. As a consequence, patient registers were established in many countries [10]. Most of these registries included only patients treated with biologic DMARDs [10], and only a few registries were designed to also include patients treated with synthetic DMARDs (e.g., the Norwegian NOR-DMARD and Danish DANBIO registries) [11, 12]. To facilitate collection of real-time data from each patient, assist clinical decision-making and improve the quality of clinical care, computer software tools have been developed [12, 13]. This has facilitated data collection and made patient benchmarking possible, and data collection for registers can be performed in one workflow [12, 14].

Despite all the register data gathered, there are limited data available reflecting the whole disease spectrum in unselected patients with RA visiting ordinary outpatient clinics. Thus, the aim of this study was to explore changes in clinical disease status and treatment in patients with RA in the first 10-year period after the monitoring all patients with RA was decided upon as a clinical standard in our rheumatology outpatient clinic.

## Methods

### Patients and data collection

In fall 2003, we decided at our rheumatology clinic to implement a clinical standard for all patients with RA to be monitored by the use of quantitative disease activity and patient-reported outcome (PRO) measures. When registration started, data were reported and collected on paper forms. In 2005, this procedure was facilitated by the implementation of a computer software program that the Hospital of Southern Norway Trust and the hospital rheumatology department had been involved in developing, the GoTreatIT Rheuma computer program (DiaGraphIT, Kristiansand, Norway) [15]. No specific protocol for tight control or any treatment protocol was used. Treatment and follow-up were based on the treating doctor’s judgment in accordance with national recommendations, and after 2007 also in accordance with the Norwegian tender system for prescription of biologic drugs.

The outpatient rheumatology clinic serves a population of approximately 290,000 inhabitants living in the two southernmost counties in Norway. Two private practice rheumatologists are practicing in the same geographic area.

The selection of outcome measures was based on recommendations given by experts in the field [7, 8]. PRO included the Modified Health Assessment Questionnaire (MHAQ) to assess physical function [16], visual analogue scales (VASs; 0–100 mm) were used to report joint pain, fatigue and patient global assessment. Morning stiffness was reported in 15-minute units.

Disease activity measures included a standardized 28 tender and swollen joint count and assessment of ankle and metatarsophalangeal joints performed by rheumatologists or by trained nurses. Laboratory markers of inflammation included C-reactive protein (CRP) and erythrocyte sedimentation rate (ESR).

The GoTreatIT Rheuma computer program was used to calculate the composite Disease Activity Score in 28 joints (DAS28)-ESR [17] and modifications of this score with the DAS28-CRP, DAS28-ESR with three variables and DAS28-CRP with three variables, the two latter without the patient global assessment reported on the VAS. In this study, we report only DAS28 scores. The assessor’s (a trained nurse or rheumatologist) global assessment of disease activity was reported on the basis of the VAS. The computer program also was used to calculate the Clinical Disease Activity Index (CDAI) [18]. Further, we calculated the Simplified Disease Activity Index (SDAI) [18]. We also registered data for rheumatoid factor (RF) and anti-citrullinated protein antibody (ACPA).

The composite scores of the DAS28, CDAI and SDAI for assessment of disease activity were also used based on cutoffs to define clinical status regarding remission, low disease activity, moderate disease activity and high disease activity. For DAS28, remission is defined as values ≤2.6, and low disease activity is defined as values >2.6 and ≤3.2, moderate disease as values >3.2 and ≤5.1 and high disease activity as values >5.1 [19]. For CDAI, remission is defined as values ≤2.8, and low disease activity is defined as values >2.8 and ≤10, moderate disease activity as values >10 and ≤22 and high disease activity as values >22. For SDAI, remission is defined as values ≤3.3, and low disease activity is defined as values >3.3 and ≤11, moderate disease activity as values >11 and ≤26 and high disease activity as values >26 [18]. We also applied the Boolean remission criteria in accordance with the new American College of Rheumatology/European League Against Rheumatism (EULAR/ACR) guidelines for remission [20].

Previous and current use of disease-modifying drugs were registered and updated systematically at all visits and included prednisolone as well as synthetic and biologic DMARDs. In 2006, we also performed a systematic review of all medical records of the patients with RA in the hospital system by retrieving data on previous use of prednisolone and synthetic and biologic DMARDs recorded since 1999. Demographic data were also registered. Starting in 2010, self-reported height and weight, smoking status, years of education and work status were included as part of standard routine data recording by use of the computer program.

### Statistical analysis

Continuous variables were presented as mean with standard deviation. Categorical variables were presented as number and percentage. To explore for a change in variables and associations over the 10-year period and the last 4-year period, we used linear regression for continuous variables and χ^2^ test for categorical variables. For categorical variables that were significant, we performed post hoc analysis to identify where the differences were between the separate years.

### Ethics

The approval for this study was given by our regional ethics committee (Regional etisk komite Midt-Norge 2010/3078). No consent from patients was needed, which was approved by the ethics committee, as all the data were collected as part of clinical care to facilitate treatment decisions.

## Results

As shown in Table 1, the number of patients with RA at our outpatient clinic with at least one annual visit for whom outcome measures were reported increased from 404 in 2004 to a maximum of 1113 patients in 2012, reaching a plateau in the last 4 years (2010–2013) of approximately 1100 patients with RA. The annual percentage of women ranged from 68.3 % to 74.0 %. Table 1 displays data for age, sex, body mass index (BMI), years of education, employment status, smoking, disease duration and RF and ACPA status.

**Table 1:** Clinical and demographic data for our study sample

Year (patients)	Female	Age (yr)	BMI (kg/m^2^)	Education (yr)	Full-time job if age <65 yr	Current smoker	Disease duration (yr)	RF+	ACPA+
2004	74.0 %	69.2 (13.8)	NA	NA	NA	NA	12.3 (10.3)	71.6 %	66.7 %
(n = 404)	[100.0 %]	[100.0 %]					[100.0 %]	[45.3 %]	[0.7 %]
2005	70.9 %	69.7 (14.8)	NA	NA	NA	NA	11.5 (11.1)	68.3 %	76.4 %
(n = 604)	[100.0 %]	[100.0 %]					[100.0 %]	[51.2 %]	[9.1 %]
2006	70.5 %	68.7 (15.0)	NA	NA	NA	NA	11.6 (11.0)	71.3 %	70.1 %
(n = 620)	[100.0 %]	[100.0 %]					[100.0 %]	[57.3 %]	[23.7 %]
2007	70.8 %	67.9 (14.6)	NA	NA	NA	NA	10.7 (10.8)	68.3 %	69.1 %
(n = 743)	[100.0 %]	[100.0 %]					[100.0 %]	[62.0 %]	[38.0 %]
2008	69.8 %	66.3 (14.3)	NA	NA	NA	NA	10.9 (10.9)	68.6 %	69.6 %
(n = 796)	[100.0 %]	[100.0 %]					[100.0 %]	[70.9 %]	[52.1 %]
2009	68.8 %	65.7 (14.1)	NA	NA	NA	NA	10.4 (10.4)	64.8 %	67.0 %
(n = 969)	[100.0 %]	[100.0 %]					[100.0 %]	[81.8 %]	[67.2 %]
2010	68.9 %	65.2 (14.0)	25.8 (4.3)	11.1 (3.4)	26.6 %	23.9 %	10.7 (10.4)	63.9 %	65.6 %
(n = 1069)	[100.0 %]	[100.0 %]	[93.0 %]	[94.4 %]	[97.1 %]	[97.1 %]	[100.0 %]	[91.8 %]	[80.5 %]
2011	68.3 %	64.9 (14.1)	25.7 (4.4)	11.2 (3.5)	22.8 %	22.7 %	10.9 (10.5)	66.1 %	68.1 %
(n = 1103)	[100.0 %]	[100.0 %]	[96.4 %]	[87.7 %]	[88.6 %]	[86.9 %]	[100.0 %]	[95.5 %]	[90.1 %]
2012	68.8 %	64.3 (14.1)	25.8 (4.5)	11.2 (3.5)	27.0 %	20.3 %	11.4 (10.5)	65.8 %	67.9 %
(n = 1113)	[100.0 %]	[100.0 %]	[97.6 %]	[97.6 %]	[98.4 %]	[97.8 %]	[100.0 %]	[97.0 %]	[93.6 %]
2013	68.6 %	63.7 (13.8)	25.7 (4.4)	11.4 (3.5)	27.5 %	20.5 %	12.1 (10.6)	66.7 %	69.7 %
(n = 1083)	[100.0 %]	[100.0 %]	[98.3 %]	[98.3 %]	[99.0 %]	[99.0 %]	[100.0 %]	[98.0 %]	[95.5 %]
*P* value^a^ 2004–2013	0.63	<0.001	0.86	0.081	<0.001	0.13	0.45	0.20	0.66
*P* value^a^ 2010–2013	0.99	0.008	0.86	0.081	<0.001	0.13	0.001	0.58	0.30

*Abbreviations: ACPA* anti-citrullinated protein antibody, *BMI* body mass index, *NA* Not available, *RF* rheumatoid factor, yr years

Left column gives number of patients monitored. The percentages within square brackets represent patients with available data

^a^χ^2^ test for categorical and linear regression for continuous variables was used to test for differences during follow-up

For sex, BMI, years of education, smoking, RF and ACPA, there were no significant differences during follow-up. However, mean age decreased significantly from 69.2 years in 2004 to 63.7 years in 2013.

For the last 4 years, the proportion of patients <65 years of age reported to have full-time jobs ranged from 22.8 % to 27.5 %. For the last 4 years, disease duration increased significantly from 10.7 years in 2010 to 12.1 years in 2013. The same pattern was seen when men and women were tested separately. Apart from RF for biologic DMARD-treated patients, no significant differences were seen between patients treated with biologics and those taking non-biologics. The proportion of RF-positive patients was significantly higher in biologic DMARD-treated patients (range 74.6 % to 89.2 %) compared with non-biologic DMARD-treated patients for the 10 years period.

When we compared women with men, we found that women were significantly younger (by 0.9–2.7 years), had a lower BMI (by 0.6–1.0 kg/m^2^), had longer education (by 0.4–0.7 years) and had longer disease duration (by 1.2–2.9 years). Having a full-time job in individuals <65 years of age was more frequently reported for men (percentages ranged from 34.9 % in 2011 to 44.4 % in 2012) than in women (percentages ranged from 17.4 % in 2011 to 20.7 % in 2013). Minor, non-significant differences between men and women were seen for smoking, RF and ACPA.

Patients taking biologic DMARDs were at all time points significantly (*P* < 0.001) younger than patients not being treated with biologic DMARDs (by 5.4 years in 2004 to 8.7 years in 2005). For BMI, a significant difference between users and non-users of biologic DMARDs was seen only for 2013 (26.1 kg/m^2^ vs. 25.5 kg/m^2^; *P* = 0.02). Patients taking biologic DMARDs had significant longer education than patients not receiving biologic DMARDs; however, this was true only for 2010–2012 (longer by 0.7–0.8 years) and not for 2013 (longer by 0.4 years). Biologic DMARD–treated patients had significantly longer disease duration than patients not treated with biologic DMARDs (longer by 2.3 years in 2005 and 3.6 years in 2010) apart from 2004 (longer by 1.3 years; P = 0.24) and 2006 (longer by 1.7 years; P = 0.06). Patients taking biologic DMARDs were more frequently RF- and ACPA-positive than patients not treated with biologic DMARDs. 

Table 2 displays disease activity data, and Table 3 shows the PRO data.

**Table 2:** Measures of disease activity for each year from 2004 to 2013 for patients with rheumatoid arthritis monitored with outcome measures in an ordinary outpatient clinic

Year (patients)	ESR (mm/h)	CRP (mg/dl)	28 tender joint count	28 swollen joint count	DAS28-ESR	CDAI	SDAI	Assessor’s global assessment (VAS, mm)
2004	22.3 (16.8)	14.8 (18.9)	4.2 (5.5)	4.1 (4.6)	3.9 (1.4)	14.8 (10.9)	16.2 (11.8)	22.6 (16.1)
(n = 404)	[77.7 %]	[81.2 %]	[87.4 %]	[87.4 %]	[74.3 %]	[85.0 %]	[77.2 %]	[86.6 %]
2005	22.1 (17.4)	13.6 (15.2)	4.0 (5.5)	3.5 (4.1)	3.8 (1.4)	13.5 (10.6)	14.7 (11.5)	19.9 (15.3)
(n = 604)	[80.8 %]	[79.8 %]	[90.2 %]	[90.2 %]	[79.6 %]	[89.4 %]	[78.5 %]	[90.1 %]
2006	22.7 (16.5)	13.0 (13.1)	4.8 (5.7)	3.3 (3.7)	4.0 (1.4)	14.4 (10.4)	15.5 (10.8)	20.1 (16.1)
(n = 620)	[76.6 %]	[78.4 %]	[90.0 %]	[90.0 %]	[75.2 %]	[87.6 %]	[76.0 %]	[88.9 %]
2007	23.3 (18.7)	14.9 (18.0)	4.5 (5.7)	3.3 (4.0)	3.9 (1.4)	13.9 (11.0)	15.7 (11.9)	21.0 (16.3)
(n = 743)	[63.4 %]	[66.8 %]	[72.1 %]	[72.1 %]	[57.9 %]	[67.3 %]	[60.4 %]	[71.3 %]
2008	20.9 (16.2)	10.9 (17.4)	3.6 (4.8)	3.1 (3.7)	3.6 (1.3)	12.2 (10.0)	13.3 (10.6)	17.9 (15.9)
(n = 796)	[62.8 %]	[66.7 %]	[74.9 %]	[74.9 %]	[56.0 %]	[64.1 %]	[58.7 %]	[71.4 %]
2009	19.4 (15.8)	8.4 (14.2)	2.9 (4.6)	2.7 (3.6)	3.3 (1.3)	10.8 (9.4)	11.9 (10.1)	17.0 (16.0)
(n = 969)	[70.6 %]	[74.0 %]	[83.4 %]	[83.4 %]	[64.0 %]	[75.9 %]	[66.3 %]	[81.8 %]
2010	19.1 (15.0)	8.0 (12.6)	2.5 (4.2)	1.9 (3.1)	3.2 (1.3)	9.1 (8.3)	9.8 (8.6)	13.0 (12.7)
(n = 1069)	[80.4 %]	[81.2 %]	[89.9 %]	[89.9 %]	[77.4 %]	[85.4 %]	[77.6 %]	[88.5 %]
2011	18.2 (14.7)	7.1 (12.0)	1.9 (3.4)	1.4 (2.5)	3.0 (1.2)	8.0 (7.4)	8.6 (7.7)	12.9 (13.4)
(n = 1103)	[80.2 %]	[84.0 %]	[90.0 %]	[90.0 %]	[78.5 %]	[87.9 %]	[81.9 %]	[90.1 %]
2012	18.0 (15.6)	7.0 (13.2)	1.5 (3.5)	1.2 (2.5)	2.8 (1.2)	7.1 (7.1)	7.8 (7.5)	10.6 (12.6)
(n = 1113)	[78.0 %]	[83.1 %]	[91.3 %]	[91.3 %]	[75.8 %]	[88.2 %]	[80.6 %]	[91.0 %]
2013	17.2 (14.2)	6.4 (10.6)	1.3 (3.0)	0.8 (1.8)	2.7 (1.1)	6.3 (6.1)	7.0 (6.3)	9.7 (11.4)
(n = 1083)	[76.8 %]	[80.4 %]	[89.8 %]	[89.8 %]	[74.1 %]	[86.1 %]	[76.6 %]	[89.6 %]
*P* value^a^ 2004–2013	<0.001	<0.001	<0.001	<0.001	<0.001	<0.001	<0.001	<0.001
*P* value^a^ 2010–2013	0.012	0.007	<0.001	<0.001	<0.001	<0.001	<0.001	<0.001

*Abbreviations: CDAI*: Clinical Disease Activity Index, *CRP* C-reactive protein, *DAS28* Disease Activity Score in 28 joints, *ESR* Erythrocyte sedimentation rate, *SDAI* Simplified Disease Activity Index, *VAS* visual analogue scale, 0–100 mm

Data are mean (SD). The percentages within square brackets represent patients with available data

^a^Linear regression used to test for differences during follow-up

**Table 3:** Patient-reported outcome measures for each year from 2004 to 2013 for patients with rheumatoid arthritis monitored in an ordinary outpatient clinic

Year (patients)	MHAQ	Joint pain (VAS, mm)	Fatigue (VAS, mm)	Mornings stiffness (h)	Patient global assessment (VAS, mm)
2004	0.57 (0.53)	40.2 (26.8)	45.7 (29.5)	1.1 (1.3)	43.4 (26.5)
(n = 404)	[96.0 %]	[88.1 %]	[86.9 %]	[90.6 %]	[88.4 %]
2005	0.58 (0.55)	39.2 (25.8)	42.9 (29.2)	1.0 (1.3)	40.9 (24.8)
(n = 604)	[95.0 %]	[90.4 %]	[89.1 %]	[92.1 %]	[90.4 %]
2006	0.61 (0.58)	38.8 (26.8)	43.4 (30.0)	1.0 (1.0)	42.8 (25.9)
(n = 620)	[96.8 %]	[91.8 %]	[91.3 %]	[92.6 %]	[90.2 %]
2007	0.60 (0.53)	38.8 (25.9)	41.2 (30.0)	1.2 (1.4)	39.1 (25.7)
(n = 743)	[94.3 %]	[90.2 %]	[90.8 %]	[91.5 %]	[79.1 %]
2008	0.56 (0.53)	36.8 (25.5)	41.4 (29.6)	1.0 (1.3)	38.2 (25.2)
(n = 796)	[90.6 %]	[86.2 %]	[89.4 %]	[89.2 %]	[80.9 %]
2009	0.50 (0.50)	35.7 (26.0)	37.5 (29.7)	1.0 (1.3)	35.8 (25.7)
(n = 969)	[92.7 %]	[90.1 %]	[91.3 %]	[92.0 %]	[83.7 %]
2010	0.50 ()	34.6 (25.3)	38.1 (29.6)	1.0 (1.3)	35.2 (25.5)
(n = 1069)	[96.0 %]	[94.0 %]	[94.4 %]	[94.8 %]	[90.4 %]
2011	0.51 (0.51)	35.0 (26.1)	38.4 (30.3)	1.0 (1.4)	35.1 (26.0)
(n = 1103)	[96.7 %]	[96.2 %]	[95.7 %]	[95.8 %]	[90.6 %]
2012	0.49 (0.51)	33.6 (25.6)	38.6 (30.1)	1.0 (1.3)	34.0 (25.9)
(n = 1113)	[96.6 %]	[96.2 %]	[95.9 %]	[95.6 %]	[91.2 %]
2013	0.50 (0.52)	32.6 (25.7)	37.4 (30.2)	1.0 (1.4)	33.9 (26.0)
(n = 1083)	[96.0 %]	[95.3 %]	[95.2 %]	[94.6 %]	[89.9 %]
*P* value^a^ 2004–2013	<0.001	<0.001	<0.001	<0.001	<0.001
*P* value^a^ 2010–2013	0.88	0.040	0.69	0.27	0.19

*Abbreviations: MHAQ* Modified Health Assessment Questionnaire, *VAS* Visual analogue scale

Data are mean (SD). The percentages within square brackets represent patients with available data

^a^Linear regression used to test for differences during follow-up

As shown in Table 2, all laboratory measures, joint scores and composite scores improved significantly over the 10-year period as well as for the last 4-year period. The same was true for men and women tested separately and for patients treated with biologic and non-biologic DMARDs tested separately.

**Fig. 1:**
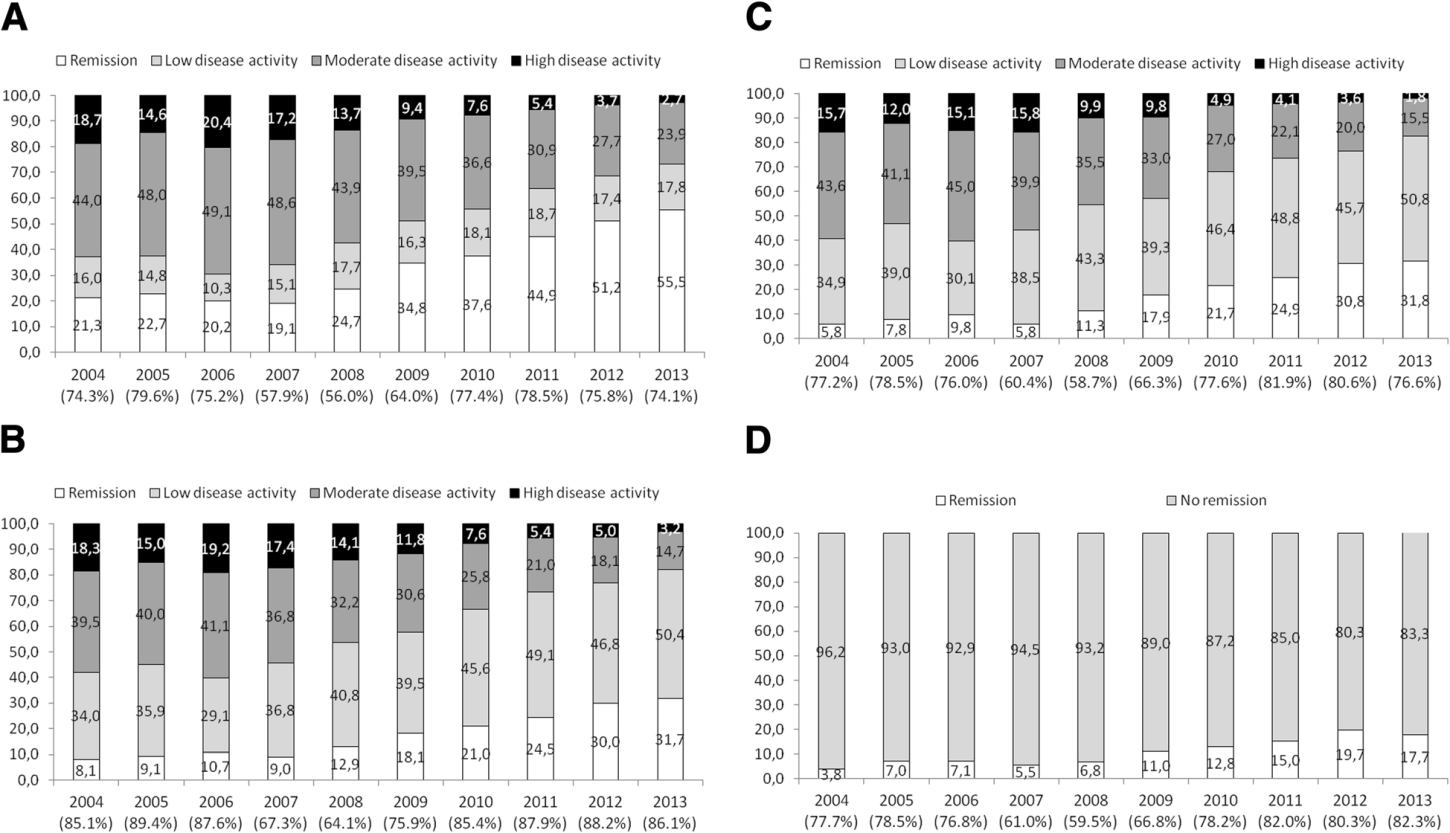
Percentage of patients with rheumatoid arthritis in remission and with low, moderate and high disease activity for each year in the 10-year period from 2004 to 2013. **a** Defined according to cutoffs for the Disease Activity Score in 28 joints [19]. **b** Defined according to cutoffs for the Clinical Disease Activity Index [18]. **c** Defined according to cutoffs for the Simplified Disease Activity Index [18]. **d** Percentages of patients in remission as defined by Boolean criteria [20]

As shown in Fig. 1, the proportions of patients in remission according to DAS28, CDAI, SDAI and Boolean assessments increased significantly from 21.3 %, 8.1 %, 5.8 %, and 3.8 %, respectively, in 2004 to 55.5 %, 31.7 %, 31.8 % and 17.7 %, respectively, in 2013. The proportions of patients with low disease activity status according to DAS28, CDAI and SDAI scores were 16.0 %, 34.0 % and 34.9 %, respectively, in 2004 and 17.8 %, 50.4 % and 50.8 %, respectively, in 2013. As shown in Table 3, PRO improved significantly over the 10-year period. However, for the last 4 years only, a minor significant improvement was seen for joint pain, whereas no significant differences were found for MHAQ, fatigue, morning stiffness or patient global assessment.

In Table 4 the proportion of patients treated with prednisolone, biologic and synthetic DMARDs or in combinations is displayed. The proportion of patients with RA who were not treated with prednisolone, biologic or synthetic DMARDs during the 10 years period was rather stable around 10 %. A high proportion of patients were over this 10 years period treated with biologic DMARDs with the lowest proportion in 2006 (26.0 %) and the highest in 2004 (32.9 %) with tumor necrosis factor (TNF) inhibitor being the most frequent used biologic drug (20.0 %–34.4 %). Among the TNF inhibitor treated patients the proportion of patients concomitantly treated with synthetic DMARDs was lowest in 2008 (68.8 %) and highest in 2011 (76.9 %). For the non-TNF biologic DMARDs there was a slight increase of 0.5 % in 2004 to 13.5 % in 2013 with rituximab being most frequently used. The proportion of patients using synthetic DMARDs was also stable in the 10 years period (58.0 %–66.1 %) with methotrexate (MTX) being the most frequent used synthetic DMARD (44.4 %–52.5 %). A rather high proportion of patients, approximately 55–60 %, were using prednisolone over the period. The proportion of patients using both synthetic and biologic DMARDs and prednisolone was rather stable for the whole period (9.2 %–13.1 %). Detailed information for the single synthetic and biologic DMARDs is depicted in Table 4.

**Table 4:** Treatment is displayed for each year in the 10-year period from 2004 to 2013 for patients with rheumatoid arthritis monitored with outcome measures in an ordinary outpatient clinic

Treatment	2004	2005	2006	2007	2008	2009	2010	2011	2012	2013	*P* value
	(n = 404)	(n = 604)	(n = 620)	(n = 743)	(n = 796)	(n = 969)	(n = 1069)	(n = 1103)	(n = 1113)	(n = 1083)	(2010–2013)
No treatment^a^	44 (10.9)	102 (16.9)	94 (15.2)	110 (14.8)	99 (12.4)	139 (14.3)	122 (11.4)	121 (11.0)	132 (11.9)	106 (9.8)	0.45
Biologic DMARDs (%)	141 (34.9)	170 (28.1)	161 (26.0)	203 (27.3)	234 (29.4)	265 (27.3)	335 (31.3)	342 (31.0)	366 (32.9)	373 (34.4)	0.30
TNF inhibitor	139 (34.4)	170 (28.1)	159 (25.6)	176 (23.7)	192 (24.1)	198 (20.4)	236 (22.1)	221 (20.0)	230 (20.7)	227 (21.0)	0.70
Non-TNF inhibitors	2 (0.5)	0 (0)	2 (0.3)	27 (3.6)	42 (5.3)	67 (6.9)	99 (9.3)	121 (11.0)	136 (12.2)	146 (13.5)	0.016
Synthetic DMARDs (%)	267 (66.1)	349 (58.8)	351 (56.6)	440 (59.2)	471 (59.2)	562 (58.0)	627 (58.7)	667 (60.5)	668 (60.0)	660 (60.9)	0.73
One synthetic DMARD (%)	243 (60.1)	321 (53.1)	342 (55.2)	423 (56.9)	455 (57.2)	547 (56.4)	612 (57.2)	648 (58.7)	649 (58.3)	649 (59.9)	0.88
Two synthetic DMARDs (%)	24 (5.9)	28 (4.6)	9 (1.5)	16 (2.2)	16 (2.0)	14 (1.4)	14 (1.3)	18 (1.6)	17 (1.5)	10 (0.9)	
Three synthetic DMARDs (%)	0 (0)	0 (0)	0 (0)	1 (0.1)	0 (0)	1 (0.1)	1 (0.1)	1 (0.1)	2 (0.2)	1 (0.1)	
Biologic and synthetic DMARDs	100 (24.8)	123 (20.4)	114 (18.4)	142 (19.1)	155 (19.5)	176 (18.2)	216 (20.2)	226 (20.5)	234 (21.0)	233 (21.5)	0.58
TNF inhibitor and synthetic DMARDs	99 (24.5)	123 (20.4)	113 (18.2)	129 (17.4)	132 (16.6)	145 (15.0)	167 (15.6)	170 (15.4)	170 (15.3)	162 (15.0)	0.94
Prednisolone (%)	224 (55.4)	334 (55.3)	369 (59.5)	433 (58.3)	486 (61.1)	589 (60.8)	653 (61.1)	653 (59.2)	617 (55.4)	590 (54.5)	0.005
Prednisolone and synthetic DMARDs (%)	152 (37.6)	208 (34.4)	216 (34.8)	270 (36.3)	297 (37.3)	354 (36.5)	377 (35.3)	394 (35.7)	368 (33.1)	346 (31.9)	0.43
Biologic DMARD and prednisolone	53 (13.1)	61 (10.1)	57 (9.2)	78 (10.5)	90 (11.3)	101 (10.4)	125 (11.7)	125 (11.3)	124 (11.1)	120 (11.1)	0.80
Biologic DMARDs											
Adalimumab (%)	27 (6.7)	50 (8.3)	51 (8.2)	56 (7.5)	58 (7.3)	80 (8.3)	78 (7.3)	70 (6.3)	61 (5.5)	52 (4.8)	0.08
Certolizumab (%)	0 (0)	0 (0)	0 (0)	0 (0)	0 (0)	0 (0)	8 (0.7)	10 (0.9)	21 (1.9)	44 (4.1)	0.000
Etanercept (%)	60 (14.9)	77 (12.7)	68 (11.0)	73 (9.8)	96 (12.1)	88 (9.1)	98 (9.2)	109 (9.9)	118 (10.6)	104 (9.6)	0.72
Golimumab (%)	0 (0)	0 (0)	0 (0)	0 (0)	0 (0)	0 (0)	26 (2.4)	14 (1.3)	12 (1.1)	9 (0.8)	0.008
Infliximab (%)	52 (12.9)	43 (7.1)	40 (6.5)	47 (6.3)	38 (4.8)	30 (3.1)	26 (2.4)	18 (1.6)	18 (1.6)	18 (1.7)	0.42
Abatacept (%)	0 (0)	0 (0)	0 (0)	3 (0.4)	3 (0.4)	5 (0.5)	11 (1.0)	21 (1.9)	23 (2.1)	15 (1.4)	0.19
Anakinra (%)	2 (0.5)	0 (0)	1 (0.2)	0 (0)	0 (0)	0 (0)	0 (0)	0 (0)	0 (0)	0 (0)	–
Rituximab (%)	0 (0)	0 (0)	1 (0.2)	24 (3.2)	39 (4.9)	58 (6.0)	76 (7.1)	88 (8.0)	102 (9.2)	118 (10.9)	0.013
Tocilizumab (%)	0 (0)	0 (0)	0 (0)	0 (0)	0 (0)	4 (0.4)	12 (1.1)	12 (1.1)	11 (1.0)	13 (1.2)	0.97
Synthetic DMARDs											
Leflunomide (%)	15 (3.7)	31 (5.1)	29 (4.7)	40 (5.4)	44 (5.5)	55 (5.7)	63 (5.9)	64 (5.8)	62 (5.6)	58 (5.4)	0.95
Methotrexate (%)	212 (52.5)	269 (44.5)	275 (44.4)	349 (47.0)	360 (45.2)	438 (45.2)	496 (46.4)	544 (49.3)	558 (50.1)	551 (50.9)	0.17
Methotrexate dose weekly (mg)	10.0 (2.3)	10.2 (2.7)	10.6 (2.6)	10.8 (2.8)	11.3 (3.0)	11.9 (3.0)	12.3 (3.4)	12.6 (3.4)	13.2 (3.6)	13.5 (3.8)	<0.001
Sulfasalazine (%)	12 (3.0)	20 (3.3)	21 (3.4)	27 (3.6)	31 (3.9)	31 (3.2)	39 (3.6)	36 (3.3)	32 (2.9)	39 (3.6)	0.73
Hydroxychloroquine (%)	37 (9.2)	42 (7.0)	24 (3.9)	30 (4.0)	35 (4.4)	39 (4.0)	36 (3.4)	35 (3.2)	31 (2.8)	20 (1.8)	0.14
Other synthetic DMARDs^b^ (%)	15 (3.7)	15 (2.5)	11 (1.8)	11 (1.5)	16 (2.0)	14 (1.4)	9 (0.8)	7 (0.6)	5 (0.4)	3 (0.3)	0.32

*Abbreviations: DMARDs* disease-modifying antirheumatic drugs, *TNF* tumor necrosis factor

Data are presented as numbers and percentages (%) or as mean with standard deviation

χ^2^ test or linear regression used to test for differences during follow-up for the period from 2010 to 2013

^a^No prednisolone, biologic or synthetic DMARDs

^b^Auranofin, azathioprine, ciclosporin and gold

## Discussion

The strength and importance of this quality study of clinical practice are the insight and information gained on disease status and treatment obtained from an unselected entire RA outpatient clinic population. The data are based on standard implementation of outcome measures as part of clinical routine care recommended by leading experts [7, 8] and endorsed by EULAR [6].

The data from our study show that both laboratory and clinical measures of disease activity significantly improved annually for the patients with RA, both for the entire 10-year period and for the last 4-year period when the number of monitored patients with RA had stabilized at around 1100 patients annually. In 2013, the proportion of patients in remission was significantly higher than in 2010, despite the fact that the percentage of patients with RA taking biologic DMARDs did not increase significantly during follow–up (range 31–34 %). For synthetic DMARDs, no significant difference was found for the last 4-year period. For the period from 2010 to 2013, when the number of monitored patients with RA had stabilized at around 1100, the proportion of patients using biologic DMARDs ranged from 31 % to 34 %, and for TNF inhibitors, the percentage was stable at approximately 20 %. For synthetic DMARDs during that 4-year period, the percentage was approximately 60 %. Interestingly the proportion of patients taking prednisolone decreased significantly from 61 % in 2010 to 55 % in 2013.

On the basis of randomized studies, it is known that a treatment strategy of tight control in patients with RA using quantitative outcome measures leads to a more favorable outcome than treatment according to routine care by doctors’ judgment alone [21]. Further data from the FIN-RACo trial indicate that physicians’ adherence to a tight control treatment strategy is important not only for achieving remission but also maintaining ability to work in patients with early RA [22]. Our data revealed that 2013 had the highest proportion of patients with RA who were working full-time in the whole 10-year period.

In the NOR-DMARD registry from 2000 to 2010, the proportion of patients with RA in CDAI remission after 6 months of MTX treatment increased from 5 % in 2000–2002 to 22 % in 2009–2010, and the proportion for DAS28 remission in the whole 10-year period increased from 18 % to 38 % [23]. The proportion of patients with RA in DAS28 and CDAI remission in our study was higher and increased from 21.3 % to 55.5 % and 8.1 % and 31.7 %, respectively, from 2004 to 2013. This may be attributed to the fact that our RA population reflects no selection of outpatient clinic patients and that our data represent an annual status description of an unselected RA outpatient clinic population. In the NOR-DMARD registry, only patients staring synthetic or biologic DMARD treatment are included. Other register data also indicated that starting DMARD treatment early and at lower disease activity levels also results in improved remission rates and reduced joint damage [23–27].

Other explanations for improvement in disease activity in our study may be that, over the years, other biologic DMARDs with modes of action other than TNF inhibition have become available for use in patients in whom TNF inhibitor therapy has failed. Further, Norway has relatively liberal guidelines for prescribing biologic DMARDs. In general, only one synthetic DMARD therapy needs to have failed before biologic DMARD treatment is started. We also should mention that the mean weekly dose of MTX in our study increased significantly during the entire period, from 10.0 mg in 2004 to 13.5 mg in 2013.

The proportion of patients with RA in Norway treated with biologic DMARDs has been shown to be among the highest in the world, together with the United States and Sweden [28]. In our study, approximately one-third of the patients with RA were treated with biologic DMARDs. A huge difference in the use of biologic DMARDs across European countries was reported in the QUEST-RA multinational study [29]. However, with decreasing prices of biologics owing to the availability of biosimilars, biologic DMARDs may reach wider affordability across countries.

A favorable clinical outcome in patients with RA may also be achieved using other aggressive treatment strategies (e.g., triple combination therapy). In a recent cross-sectional report comparing our RA patient cohort with a Finnish RA outpatient cohort using approximately 50 % less biologic DMARDs than in our cohort, an even more favorable outcome was found for measures of disease activity and PRO measures [30]. In this cohort, however, 37 % were using triple combination therapy (MTX, sulfasalazine and hydroxychloroquine), as compared with 0.8 % in Norway.

The distribution of biologic DMARDs used in our outpatient clinic in the various years is reflected not only by clinical indications and doctors’ preferences but also by the Norwegian tender system. In Norway every year since 2008, vendors of biologic DMARDs give a price offer for their drugs, which results in a hierarchical list where the cheapest drug is expected to be used if no other reasons are present.

The pattern of improvement during follow-up that we saw for measures reflecting disease activity was not seen for PRO measures. For PRO measures, improvement was seen only for the whole 10-year period; no significant improvement was seen for the last 4 years, apart for joint pain, for which only a minor significant improvement was observed.

An intrusive question that emerges as more and more patients are in clinical remission is whether patients in remission should be monitored with more sensitive imaging methods to detect inflammation, such as ultrasound [31]. To date, no data exist that indicate whether ultrasound-guided decision-making in clinical care will improve clinical and radiographic outcomes in patients with RA. The aim of the ongoing Aiming for Remission in Rheumatoid Arthritis, or “ARCTIC,” study (ClinicalTrials.gov Identifier: NCT01205854) is to clarify whether the use of ultrasound in clinical care will lead to better care for patients with RA.

Our study also reflects the challenges of implementing outcome measures in clinical practice. Despite the decision at our department in 2003 to monitor all RA outpatient clinic patients with feasible measures, it took 6 years until this strategy was successfully implemented, reaching a plateau of approximately 1100 patients annually in the last 4 years of the 10-year period. The main obstacle in our experience is related to convincing health care professionals to work and communicate with the use of outcome measures. Ongoing education, reminders and feedback to nurses and doctors seems to be important and is reflective of the nature of this continuous work toward quality improvement.

The implementation of a computer system tailored for our clinic has been of great importance and value, facilitating the implementation of outcome measures in RA as part of standard clinical care. This is supported by experiences with the successful DANBIO registry, which is designed to capture operational data as part of routine clinical care and also provides a benchmarking system for the rheumatologist in clinical practice [12]. Data from each visit in the DANBIO system are stored as a sort of electronic patient file. The computer system we used (GoTreatIT Rheuma software) is designed as a clinical benchmarking system and is now also used as a data collection tool for NOR-DMARD [14] and will also be used in the new national arthritis registries in Norway (NorArtritt) and Finland.

In our experience, the computer program, which allows patients to report their clinical status using feasible PROs directly into the computer system before consultation, and the documentation of disease status and treatment directly in the system as well as visualization of the results (e.g., on time view graphs and by using joint count) have facilitated the implementation of clinical standard monitoring patients with RA. In our and others’ experience, the time cost of the clinical assessments for the physician is regained by the time saved with the availability of useful, structured and reliable data [12].

For demographic data (e.g., age, sex distribution, BMI, years of education), disease duration, prevalence of RF and ACPA were comparable to the data in other reports (e.g., the multinational QUEST-RA study) [29]. The proportion of patients with RA in our cohort who were current smokers was 20–24 %, whereas in the large, multinational QUEST-RA database, 15 % of patients with RA were reported to be current smokers; however, there were large differences between participating centers, ranging from 8 % to 56 % in men and from 1 % to 32 % in women [32].

The internal validity of our study is judged to be good because the vast majority of patients with RA have been registered. Whether our data can be extrapolated to other outpatient clinics is an open question, even when they are compared with other departments in Norway. In the future, the Norwegian national arthritis register (NorArtritt) will illuminate this question. However, our approach allows patient benchmarking and data collection for patient registries to be performed in one workflow. Our study has obvious limitations. In a rather large proportion of patients with RA, the number of patients with missing data (e.g., DAS28) was for some years rather high. Further, the lack of standardization of follow-up may also have influenced the outcome; however, it is reflective of real-life clinical situations. Unfortunately we do not have radiographic joint damage data, which are of importance for assessing long-term outcomes in patients with RA.

## Conclusions

Our real-life data, obtained from monitoring all patients with RA in an ordinary outpatient clinic, reflect the dramatic improvement in clinical outcomes in patients with RA that has occurred in the new millennium. Data reflecting the entire RA population in an outpatient clinic are of special interest and importance to understanding the heterogeneity and severity of disease activity as well as PRO in patients with RA. Such data are also important for health care planning related to the management of patients with RA.

## Abbreviations

ACPA: Anti-citrullinated protein antibody
ACR: American College of Rheumatology
BMI: Body mass index
CDAI: Clinical Disease Activity Index
CRP: C-reactive protein
DAS: Disease Activity Score
DMARD: Disease-modifying antirheumatic drug
ESR: Erythrocyte sedimentation rate
EULAR: European League Against Rheumatism
MHAQ: Modified Health Assessment Questionnaire
MTX: Methotrexate
NA: Not available
PRO: Patient-reported outcome
RA: Rheumatoid arthritis
RF: Rheumatoid factor
SDAI: Simplified Disease Activity Index
TNF: Tumor necrosis factor
VAS: Visual analogue scale
